# Increased Prevalence of Alpha-1-Antitrypsin Deficiency in Patients with Biliary Tract Cancer and Its Associated Clinicopathological Features

**DOI:** 10.3390/cells12121663

**Published:** 2023-06-19

**Authors:** Martin Cornillet, Helen Zemack, Hannes Jansson, Ernesto Sparrelid, Ewa Ellis, Niklas K. Björkström

**Affiliations:** 1 Center for Infectious Medicine, Department of Medicine Huddinge, Karolinska Institutet, Karolinska University Hospital, SE-14186 Stockholm, Sweden; 2 Division of Transplantation Surgery, Department of Clinical Science, Intervention and Technology, Karolinska Institutet, Karolinska University Hospital, SE-14186 Stockholm, Sweden; 3 Division of Surgery and Oncology, Department of Clinical Science, Intervention and Technology, Karolinska Institutet, Karolinska University Hospital, SE-14186 Stockholm, Sweden

**Keywords:** alpha-1 antitrypsin deficiency, biliary tract cancer, survival

## Abstract

Alpha-1 antitrypsin deficiency (A1ATD) is underdiagnosed and associated with liver diseases. Here, we genotyped 130 patients with biliary tract cancer (BTC) scheduled for liver resection and found A1ATD in 10.8% of the patients. A1ATD was found in all BTC subtypes, and patients had similar clinical features as non-A1ATD BTC, not permitting their identification using clinical routine liver tests. In intrahepatic cholangiocarcinoma (iCCA), the abundance of A1AT protein was increased in the tumor and appeared to be influenced by the genomic alterations. On the one hand, BTC with A1ATD had lower perineural invasion at histopathology and displayed a longer survival, suggesting that a deficiency in this protein is associated with a less aggressive phenotype. On the other hand, iCCA with high A1AT expression had more advanced tumor staging and enriched pathways for complement system and extracellular matrix interactions, indicating that A1AT protein might contribute to a more aggressive phenotype. With increased awareness, screening, and basic studies, A1ATD could represent one more layer of stratification for future targeted therapies in BTC.

## 1. Introduction

Case series report a co-occurrence of cholangiocarcinoma and alpha-1-antitrypsin (A1AT) deficiency, suggesting an underlying pathological mechanism [1,2]. Biliary tract cancer (BTC) such as cholangiocarcinoma is rare, often diagnosed at a late stage, poorly understood, and highly fatal [3]. Efforts are currently underway to develop and implement personalized therapies in BTC. However, for these to be effective, a better understanding of disease pathogenesis and the identification of subgroups of patients are needed. A1AT deficiency is an under-diagnosed inherited autosomal co-dominant disorder mostly known to be associated with chronic obstructive pulmonary disease (COPD), but also with various chronic liver diseases [4,5]. A1AT is synthetized and then secreted mainly by hepatocytes and plays an important role during inflammatory processes by inhibiting serine proteases, particularly neutrophil elastase, which is known to play a role in tumor initiation, growth, and metastatic processes [6]. Allelic variants (commonly referred to as M, S, and Z) of the SERPINA1 gene encoding A1AT influence its production, secretion, and functional activity. In the case of A1AT deficiency, leading to liver diseases, A1AT mutated protein would polymerize, aggregate, and accumulate in the endoplasmic reticulum of hepatocytes, leading to cell death, chronic liver injury, and/or carcinogenesis, possibly affecting the biliary tract within the liver. Here, we describe the prevalence of A1AT deficiency in a large, well-characterized cohort of patients with various types of BTC, and its relationship with the clinical and biological phenotypes of the patients.

## 2. Materials and Methods

### 2.1. Genotyping and qPCR

Genomic DNA was isolated from whole blood using Gentra Puregene Blood Kit (Qiagen, Venlo, The Netherlands). Single Nucleotide Polymorphism (SNP) genotyping was used on all included human samples. Allelic discrimination method was performed using TaqMan^®^ assays on an ABI Step-One Plus (Applied Biosystems, Foster City, CA, USA) real-time PCR instrument according to manufacturer’s instruction. Pre-designed SNP Genotyping Assay probes were purchased from Applied Biosystems. PiS allele, rs1758 (assay ID: C_594695_20); PiZ allele, rs28929474 (assay ID: C_34508510_10); PiM2/M4 allele, rs709932 (assay ID: C_2895146_20) PiNull allele, rs28929473 (assay ID: C_63321235_20). SNP analysis PCR was performed on an ABI Step-One Plus instrument.

### 2.2. Cohort

Consecutive patients that underwent surgical exploration with a diagnosis of perihilar cholangiocarcinoma (pCCA), intrahepatic cholangiocarcinoma (iCCA), or gallbladder cancer (GBC), confirmed postoperatively by pathology, at Karolinska University Hospital (Stockholm, Sweden), a tertiary referral center, from January 2009 until January 2017 were assessed for inclusion. Data was retrospectively collected from electronic health records. The study was approved by the Swedish Ethical Review Authority (2013/188-31/1). One out of 14 patients with A1ATD (7%) and 4 out of 126 patients without A1ATD (3%) had cirrhosis according to preoperative clinical diagnosis or postoperative histopathological assessment of the resected specimen. Median follow up time was 47 and 19 months, respectively for BTC with and without A1ATD. Five patients received adjuvant chemotherapy (all without A1ATD). 

### 2.3. Public Datasets

Proteomic datasets publicly available from two studies were used. The first from Carpino et al. contained data from 10 iCCA and matched non-tumoral liver tissue. The second dataset from Dong et al. initially contained data from 214 iCCA tissue. We excluded liver fluke and HBsAg positive patients or those receiving adjuvant therapy based on available metadata and performed the analysis using data from the remaining 93 patients. For both datasets, we used the normalized data provided without further transformation. 

### 2.4. Statistical Analysis

Mann–Whitney U (Prism 9) and Chi-square (Medcalc) tests were used to compare independent groups and Wilcoxon test for matched samples. The survival analysis (cox regression) was performed using Prism9 (9.5.1). Intra hospital deaths were excluded and DFS analysis performed only with resected patients. iCCA with low and high A1AT expression were defined using median A1AT protein level. Other protein and phosphoprotein abundances in these two groups were compared using Mann–Whitney U test and false discovery rate (FDR) adjusted *p*-values. Proteins reaching FDR adjusted *p*-values below 0.01 were used to perform pathway enrichment analysis using Kyoto Encyclopedia of Genes and Genomes (KEGG) as a reference. 

## 3. Results

### 3.1. Increased Prevalence of Alpha-1-Antitrypsin Deficiency in Patients with Biliary Tract Cancer

**Figure 1:**
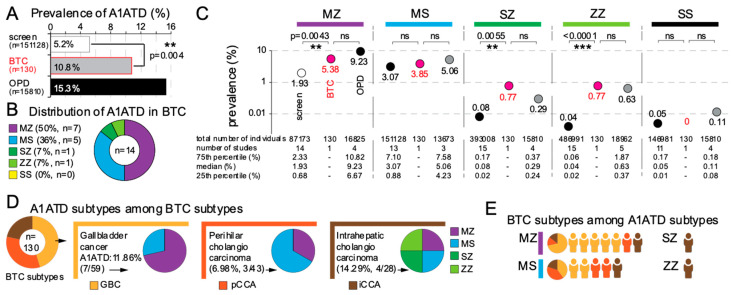
Increased prevalence of alpha-1-antitrypsin deficiency in patients with biliary tract cancer. (**A**) Prevalence of alpha-1 antitrypsin deficiency (A1ATD) in population screens of patients with biliary tract cancer (BTC) and obstructive pulmonary disease (OPD). Distribution of A1ATD in all BTC (**B**) as compared to the screening population and OPD (**C**). (**D**) Distribution of A1ATD in all BTC subtypes, perihilar cholangiocarcinoma (pCCA), intrahepatic cholangiocarcinoma (iCCA), or gallbladder cancer (GBC), and A1AT subtypes among those (**E**). ** indicates *p* < 0.01, *** indicates *p* < 0.001.

We first evaluated the prevalence of A1ATD in 130 patients with BTC scheduled for liver resection by genotyping peripheral blood DNA for M, S, and Z SERPINA1 allelic variants. We compared our results to retrospective datasets [7], including 17 studies evaluating the prevalence of A1ATD in the general population (n = 489.294 individuals in total), and four studies in patients with obstructive pulmonary disease (OPD, n = 18.962 individuals in total). We found that 10.8% (14/130) of patients with BTC had A1ATD (Figure 1A), which was twice that compared to the general population (5.2%, significantly higher, *p* = 0.004), although not as prevalent as in patients with OPD (15.3%). The majority of A1ATD genotypes found in BTC were MZ and MS (Figure 1B). Compared to the general population, the prevalence of MZ, SZ, and ZZ was significantly higher in BTC, whereas MS and SS were not (Figure 1C). We then wondered if A1ATD genotypes were associated with BTC subtypes. We found that gallbladder cancer (GBC) trended to be more associated with MZ, whereas the intrahepatic cholangiocarcinoma (iCCA) group contained one patient of each genotype (Figure 1D). Looking at the BTC subtypes per A1AT genotype, four out of seven MZ patients had GBC, whereas patients with all BTC subtypes were represented in the MS group (Figure 1E).

### 3.2. Comparative Clinicopathological and Survival Features of BTC Patients with and without A1ATD

**Figure 2:**
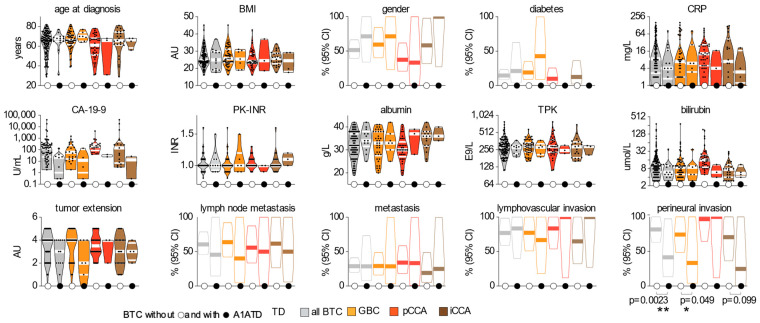
Comparative pathobiological features of BTC patients with and without A1ATD. Clinical and biological phenotypes of BTC patients with and without A1ATD. Medians and interquartile ranges are depicted in white inside the violin plot for continuous and discrete parameters. Percentages and 95% confidence intervals are depicted for binary parameters. BMI, body mass index. CRP, C-reactive protein. CA 19-9, cancer associated antigen 19-9. PK-INR, prothrombin complex, INR. TPK, platelet count. ** indicates *p* < 0.01, * indicates *p* < 0.05.

We next asked if BTC with A1ATD harbored any specific clinical or pathological features (Figure 2). No significant differences were observed with respect to age, BMI, gender, or diabetes prevalence in these patients. Additionally, they exhibited similar levels of inflammatory biomarkers, tumor biomarkers, and liver function tests since CRP, CA-19-9, PK-INR, albumin, TPK, and bilirubin did not differ significantly. Looking at tumor features, we found that BTC patients with A1ATD had similar TNM staging and lymphovascular invasion but a significant lower perineural invasion (Figure 2), possibly suggesting a distinct pathobiology. 

**Figure 3:**
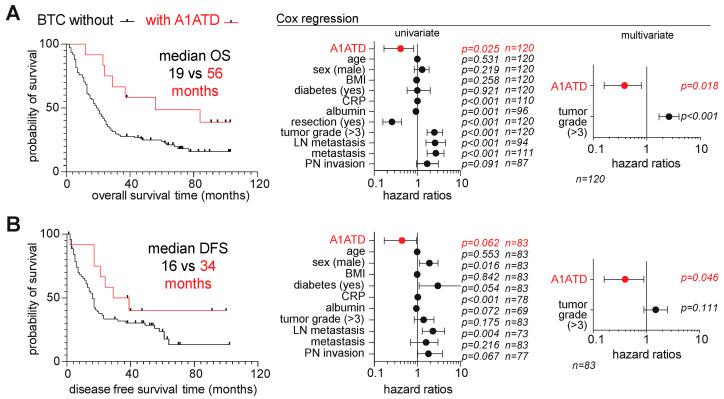
Comparative survival analysis of BTC patients with and without A1ATD. Cox regression analysis of (**A**) overall survival (OS) and (**B**) disease-free survival (DFS). LN, lymph node. PN, perineural.

We next investigated whether BTC patients with A1ATD present a differential survival. Their median survival time was 56 months, as compared to 19 months for BTC without A1ATD. Cox regressions were performed to assess the association of A1ATD with overall survival (OS) (Figure 3A) and disease-free survival (Figure 3B). In univariate analysis, A1ATD was associated with longer OS (*p* = 0.018) and DFS (*p* = 0.062). Moreover, multivariate analysis indicated that A1ATD was an independent predictor as compared to tumor grade.

### 3.3. Increased Expression of A1AT in Intrahepatic Cholangiocarcinoma and Associated Biological Pathways

**Figure 4:**
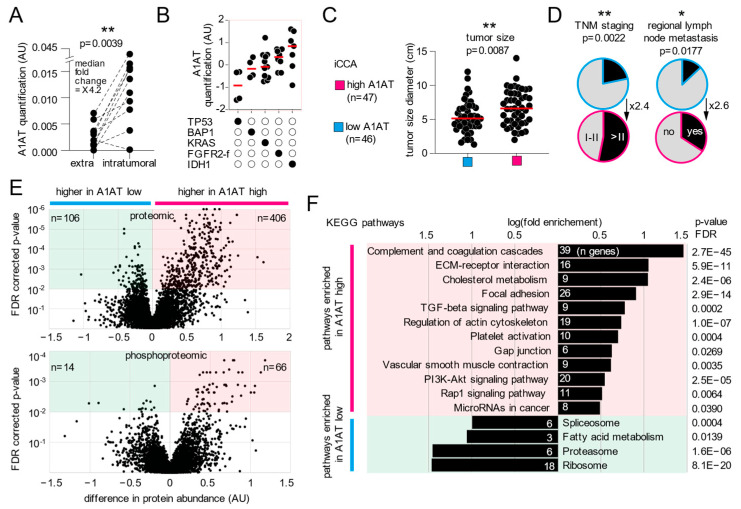
*Increase expression of A1AT in intrahepatic cholangiocarcinoma and associated biological pathways.* (**A**) Quantification of A1AT protein inside the iCCA tumor and matched surrounding liver tissue. (**B**) Quantification of A1AT protein inside the iCCA tumor in relation to the presence (black circles) or absence (white circles) of mutations in the TP53, BAP1, KRAS, and IDH1 genes, and FGFR2 fusion. AU, arbitrary unit. (**C**) Tumor size and (**D**) staging of iCCA with low or high A1AT expression. (**E**) Proteins and phosphoproteins associated with low or high A1AT expression in iCCA and corresponding enriched pathways (**F**). ** indicates *p* < 0.01, * indicates *p* < 0.05.

To explore the role of A1AT in BTC, we analyzed a recently released proteomic dataset in iCCA. Using protein quantification by Carpino et al. [8] from ten iCCA patients, we first show that the A1AT protein was four-fold enriched in CCA tumors as compared to matched non-tumoral liver tissue (Figure 4A). We then turned to a larger dataset containing only intra-tumoral protein quantifications to study A1AT protein levels inside iCCA (Dong et al. [9], 93 iCCA patients, HBsAg and liver fluke negative, naïve from adjuvant therapy). Importantly, we did not observe any correlation between SERPINA1 RNA expression and A1AT protein quantification (CI95% r = −0.3379 to 0.07354), stressing the importance of proteomic studies in CCA. Interestingly, we found that A1AT intratumoral protein expression might be modulated in the context of TP53, KRAS, IDH1, BAP1 mutations, and FGFR2 fusion (Figure 4B) and thus possibly participate in the distinct activated pathways described by Dong et al. in iCCA harboring these alterations. Next, to decipher the role of A1AT in the tumor, we compared the features of iCCA with high or low A1AT expression. This analysis revealed that iCCA with higher A1AT had larger tumor size (Figure 4C), advanced staging (2.4-fold increase of >T2), and increased lymph node metastasis (2.6-fold increase) (Figure 4D). To better understand which biological pathways might underline such features, we identified proteins and phosphoproteins in higher abundance in A1AT low and high iCCA (Figure 4E, Supplementary Table S1) and integrated those into KEGG pathway enrichment analysis. This showed that iCCA with high A1AT abundance had enriched oncogenic pathways such as complement and coagulation cascades [10], extracellular matrix interactions, TGF-beta signaling, and microRNAs, among others. Altogether, these results suggest that A1AT protein expression associates with biological pathways involved in a more aggressive tumor phenotype.

## 4. Discussion

Here, we provide a comprehensive report comparing the prevalence of A1ATD in GBC, pCCA, and iCCA. We estimate A1ATD prevalence to be around 12% in GBC and confirm an expected increase in iCCA as compared to control populations [2]. Although the relative increase in prevalence might be a matter of debate, since it depends on the type of population screened, the techniques used, and the geographical areas sampled, the proportion of patients with BTC having A1ATD is in the range of 7 to 15%, depending on the BTC subtype. These results would need to be confirmed in a larger multi-center setting, and possibly cross-analyzed with the current state-of-the-art on the natural history and classification of BTC to gain more insight into related pathobiological associations [11]. As therapies are progressing both for BTC [12,13] and A1ATD [14], a proportion of 7–15% of patients might be of clinical relevance and could, for instance, represent an even larger group than those eligible today for targeted therapies for BRAF mutation (<5%) [12,13]. Of note, how patients with BTC and A1ATD respond to approved therapies and whether they exhibit a specific mutational profile remain largely unexplored [15]. As A1ATD might affect hepatocyte homoeostasis, liver function and its regenerative response, such knowledge might be valuable when managing patients with hepatotoxic medications or intended curative surgery involving extended resections [16,17,18,19,20]. Finally, an increased peripheral level of AAT was reported to be associated with metastasis and poor prognosis in various cancers, including hepatocellular carcinoma, and thus might have a potential interest in pre-operative settings in BTC [21,22,23]. Therefore, these results might have implications for the development of new management strategies. To investigate such possibilities, retrospective screens for A1ATD using either blood or serum from existing biobanks could be performed [7].

We describe the biological, pathological, and survival profiles of BTC subtypes with and without A1ATD. Although statistical power was limited due to the relatively low number of patients in each subgroup, it was deemed sufficient to investigate the presence or absence of more considerable differences. Here, we identify reduced perineural invasion as a potential hallmark of BTC with underlying A1ATD. This finding should be validated in independent, larger cohorts but could suggest a different pathogenesis and/or clinical outcome in these patients. Although even the basic mechanisms driving perineural invasion are still poorly understood, their clinical and therapeutical relevance in BTC is starting to be appreciated [24]. One could speculate that the reduced perineural invasion in BTC with A1ATD could underline a less aggressive tumor phenotype and a better survival rate, as observed in this series.

Mechanistically, moving beyond association and investigating causality remain difficult, mainly due to a lack of basic understanding of the role of the A1AT protein, both in normal biliary tract system homeostasis and in cancer progression. We show an increased level of the A1AT protein in iCCA, modulated by the mutational profile and associated with biological pathways. Among those, complement and coagulation cascades [10], extracellular matrix interactions [25], TGFb signaling [26], and miRNAs [27,28] are important players in carcinogenesis and immune escape that might leverage potential therapies, some of which are currently evaluated in clinical trials. These results might suggest that the A1AT protein is part of a functional network of a more aggressive BTC phenotype that could be repressed by its deficiency. Small studies from the 1980s suggest that the A1AT protein might play a homeostatic role in epithelial cells from the biliary tract, not only in cholangiocytes but also in the gallbladder [29,30,31,32,33]. Thus, one could speculate that an imbalance in such homeostasis due to A1ATD could initiate a specific carcinogenic process in susceptible individuals. Several causality levels have been suggested, including a direct carcinogenesis effect of A1ATD [34], a direct priming effect followed by the acquisition of driver mutations [15], or an indirect effect by a lack of inhibition of neutrophile elastase [35]. However, larger studies using cutting-edge technologies are needed to first shed light on important homeostatic mechanisms of the A1AT protein in the biliary tract system, and then help understand its potential contribution to carcinogenic processes in this organ system.

Overall, we found that alpha-1-antitrypsin (A1AT) deficiency was present in 10.8% of patients with biliary tract cancer (BTC), a higher rate than the general population, and seemed to be associated with specific clinicopathological features. With increased awareness, screening, and basic studies, A1ATD could represent one more layer of stratification for future targeted therapies in BTC.

## Data Availability

Public datasets used in the study are available as the Supplementary Material in Carpino et al. [8] and Dong et al. [9].

## Supplementary Materials

The following supporting information can be downloaded at: https://www.mdpi.com/article/10.3390/cells12121663/s1, Table S1: proteins and phosphoproteins in higher abundance in A1AT low and high iCCA
